# Longitudinal Assessment of Cytokine Expression and Plasminogen Activation in Hantavirus Cardiopulmonary Syndrome Reveals Immune Regulatory Dysfunction in End-Stage Disease

**DOI:** 10.3390/v13081597

**Published:** 2021-08-12

**Authors:** Peter Simons, Yan Guo, Virginie Bondu, Susan L. Tigert, Michelle Harkins, Samuel Goodfellow, Cana Tompkins, Devon Chabot-Richards, Xuexian O. Yang, Laura Gonzalez Bosc, Steven Bradfute, Daniel A. Lawrence, Tione Buranda

**Affiliations:** 1 Department of Pathology, University of New Mexico School of Medicine, Albuquerque, NM 87131, USA; psimons@salud.unm.edu (P.S.); vbondu@salud.unm.edu (V.B.); cmtompki@bidmc.harvard.edu (C.T.); dchabot-richards@salud.unm.edu (D.C.-R.); 2 Bioinformatics Shared Resource Center, Division of Molecular Medicine, Department of Internal Medicine, University of New Mexico Comprehensive Cancer Center, University of New Mexico Health Sciences Center, Albuquerque, NM 87131, USA; YaGuo@salud.unm.edu; 3 Clinical and Translational Science Center (CTSC), University of New Mexico Health Sciences Center, Albuquerque, NM 87131, USA; stigert@salud.unm.edu; 4 Division of Infectious Diseases, Department of Internal Medicine, School of Medicine, University of New Mexico, Albuquerque, NM 87131, USA; MHarkins@salud.unm.edu (M.H.); SGoodfellow@salud.unm.edu (S.G.); SBradfute@salud.unm.edu (S.B.); 5 Molecular Genetics and Microbiology, University of New Mexico Health Sciences Center, Albuquerque, NM 87131, USA; XYang@salud.unm.edu; 6 Vascular Physiology Group, Department of Cell Biology and Physiology, University of New Mexico Health Sciences Center, Albuquerque, NM 87131, USA; lgonzalezbosc@salud.unm.edu; 7 Division of Cardiovascular Medicine, Department of Internal Medicine, University of Michigan Medical School, Ann Arbor, MI 48109, USA; dlawrenc@med.umich.edu

**Keywords:** PAI-1, orthohantavirus, hantavirus, principal component analysis, plasminogen activation, cytokines, ECMO, uPA, correlation, pathogenesis, cell barrier function, co-infection

## Abstract

Pathogenic New World orthohantaviruses cause hantavirus cardiopulmonary syndrome (HCPS), a severe immunopathogenic disease in humans manifested by pulmonary edema and respiratory distress, with case fatality rates approaching 40%. High levels of inflammatory mediators are present in the lungs and systemic circulation of HCPS patients. Previous studies have provided insights into the pathophysiology of HCPS. However, the longitudinal correlations of innate and adaptive immune responses and disease outcomes remain unresolved. This study analyzed serial immune responses in 13 HCPS cases due to Sin Nombre orthohantavirus (SNV), with 11 severe cases requiring extracorporeal membrane oxygenation (ECMO) treatment and two mild cases. We measured viral load, levels of various cytokines, urokinase plasminogen activator (uPA), and plasminogen activator inhibitor-1 (PAI-1). We found significantly elevated levels of proinflammatory cytokines and PAI-1 in five end-stage cases. There was no difference between the expression of active uPA in survivors’ and decedents’ cases. However, total uPA in decedents’ cases was significantly higher compared to survivors’. In some end-stage cases, uPA was refractory to PAI-1 inhibition as measured by zymography, where uPA and PAI-1 were strongly correlated to lymphocyte counts and IFN-γ. We also found bacterial co-infection influencing the etiology and outcome of immune response in two cases. Unsupervised Principal Component Analysis and hierarchical cluster analyses resolved separate waves of correlated immune mediators expressed in one case patient due to a sequential co-infection of bacteria and SNV. Overall, a robust proinflammatory immune response, characterized by an imbalance in T helper 17 (Th17) and regulatory T-cells (Treg) subsets, was correlated with dysregulated inflammation and mortality. Our sample size is small; however, the core differences correlated to survivors and end-stage HCPS are instructive.

## 1. Introduction

Sin Nombre virus (SNV) causes hantavirus cardiopulmonary syndrome (HCPS), with 30–50% case fatality rates. HCPS is characterized by loss of pulmonary vascular endothelial integrity, resulting in massive, acute pulmonary edema [1,2,3,4,5,6,7,8]. The disease progresses through different phases of severity depending on the patient. HCPS progresses rapidly, where most hospital admissions occur 3–6 days after the inception of symptoms, with most fatal outcomes occurring within 0–3 days of hospital admission. Extracorporeal membrane oxygenation (ECMO) treatment is required for most severe cases [9,10]. The virus mainly targets vascular endothelial cells without causing any visible cytopathic effects [2,11].

SNV infections occur through the airborne route to the lungs, where they are taken up by alveolar mononuclear phagocytes, such as dendritic cells (DCs) [12,13]. Productive infection of DCs protects the virions from an emergent immune response. It thus enables viruses to multiply and disseminate throughout the vascular endothelium and establish a secondary viremia before the onset of a robust host immune response [13]. Recognition of viral pathogens by Toll-like receptors expressed on innate immune cells directs the migration of antigen-presenting cells (APCs) into the draining lymph nodes (LNs) of the infected tissue site. Inside the draining LN, the APCs transmit molecular recognition information about the virus to the naive T cells while secreting T-cell stimulating cytokines.

Furthermore, priming of DCs at the site of infection enables the APCs to imprint the transformed T cells with a multi-functional program of retracing the APC’s route to the tissue-specific site of infection [14,15,16,17,18]. In this way, significant deposits of cytokine-secreting cells of myeloid and lymphoid lineage have been found in autopsied lungs, lymphoid organs, and liver tissue of HCPS decedents [2,19]. Thus, it has long been established that immune-mediated mechanisms induced by infected endothelial cells are central to the pathogenesis of SNV infections [1,2,11,12,19,20,21,22,23,24,25,26,27]. However, some animal studies have yielded conflicting evidence of the role of immune cells. For example, in one study using non-human primates [22], T-cell effector function was shown to be a significant contributor to pathology. However, elsewhere, T-cell depletion did not affect disease progression in a Syrian hamster model [28]. Thus, the role of various cells and the immune response is not well understood.

Our recent studies have shown that end-stage HCPS patients experience a significant rise in plasminogen activator inhibitor type 1 (PAI-1) within 24 h of admission [29]. Significant increases in PAI-1 expression are linked with poor outcomes in various diseases related to ischemic cardiovascular events, fibrosis, and cancer [30,31,32,33,34,35]. In addition, impaired fibrinolysis resulting from high plasma PAI-1 [34,36] can lead to excessive fibrin accumulation within vessels, such as hyaline membranes associated with HCPS [2,37]. PAI-1 is a potent inhibitor of plasminogen activators, tissue plasminogen activator (tPA), urokinase-type plasminogen activator (uPA), and plasmin. PAI-1 inhibits plasmin generation by preventing tPA or uPA from processing plasminogen to plasmin [34]. tPA is primarily involved in the dissolution of fibrin and does not appear to be a significant effector of HCPS pathogenesis [37]. uPA is synthesized as a single-chain zymogen (scuPA), also known as pro-uPA [38], and activated upon binding to urokinase-type plasminogen activator receptor (uPAR). Activated pro-uPA cleaves the zymogen plasminogen to produce the protease plasmin, converting pro-uPA to its active two-chain form [39]. PAI-1 efficiently inhibits uPA activity in plasma and most tissues by binding to both free uPA and uPAR-bound active uPA but poorly interacts with the pro-uPA-uPAR complex [40,41,42,43,44].

The uPA-uPAR system bridges the functional gap between the plasminogen activating system and inflammation by directing the migration of T cells and antigen-presenting cells, such as dendritic cells (DCs), monocytes, and macrophages, to sites of infection [41,42,43,44,45]. IFN-γ and TNF induce a plasmin-activated two-chain form of uPA [39], which binds PAI-1 with high affinity. However, IFN-γ is known to cause the tandem secretion of pro-uPA and uPAR, which then forms the pro-uPA/uPAR but binds to PAI-1 with five-fold less affinity [40] relative to the two-chain form [46]. In a previous study, we showed that HCPS patients with accompanying lymphocytosis were likely to present refractory uPA activity despite the high plasma concentration of active PAI-1 [37]. In this study, we explored longitudinal analysis of innate and adaptive cytokine expression. We identified correlations involving viral load, a robust innate and adaptive immune response; lymphocytosis; and PAI-1 refractory uPA activity, Th17/Treg imbalance, and severity of HCPS.

## 2. Materials and Methods

### 2.1. Patients

This retrospective study included 13 patients with HCPS and five healthy controls. Blood samples were originally collected in BD Vacutainer EDTA tubes from consenting patients for up to 5 consecutive days for HCPS patients and one day for healthy controls. The plasma samples were de-identified and used under UNM IRB#15-166. The patients were stratified according to severity scores: Class I (mild; n = 2, 50% male, age 32.3 ± 11.3, BMI 25.6 ± 5.9), Class II (severe; required extracorporeal membrane oxygenation (ECMO) intubation and survived, n = 6, 50% male, age 38.8 ±22.8 BMI 27.6 ± 6.2), and Class III (terminal cases; n = 5, 40% male, age 42 ± 20.5, BMI 30.2 ± 8). We also conducted a retrospective powerchart review of 52 HCPS patients’ clinical records of 15 Class I, 22 Class II, and 15 Class III under UNM IRB #16-084.

### 2.2. Analyses of 23 Cytokines

Plasma concentrations of interleukin (IL)-1α, 1β, 2, 4, 6, 10, 12p40, 12p70, 13, 15, 18, 21, 22, 25, Tumor Necrosis Factor α (TNF-α), interferon (IFN)-α,-β,-γ, Granulocyte-macrophage colony-stimulating factor (GM-CSF), Transforming Growth Factor Beta 1 (TGF-β1), monocyte chemoattractant protein-1 (MCP-1/CCL2), FMS-like tyrosine kinase 3 ligand (FLT3-L), and Granulocyte-colony-stimulating factor (G-CSF) were measured in duplicate using a custom LXSAHM Human Magnetic Luminex Assay kit from R&D Systems using a Bio-Rad Bioplex 200, following a protocol provided by the manufacturer.

### 2.3. Analyses of Plasma Levels of uPA and PAI-1

Active uPA (Catalog # HUPAKT; https://mol-innov.com/files/kis/HUPAKT.pdf accessed on 18 June 2021), total uPA (HUPAKT-TOT; https://mol-innov.com/files/kis/HPAIKT-TOT.pdf accessed on 18 June 2021), tPA (HTPA; https://mol-innov.com/files/kis/HTPAKT.pdf accessed on 18 June 2021) and active PAI-1 (HPAIKT; https://mol-innov.com/files/kis/HPAIKT.pdf accessed on 18 June 2021) were measured by ELISA kits (Molecular Innovations, Novi, MI, USA), following protocols supplied by the manufacturer at the respective web addresses shown here for each product. The website links were last accessed on 9 August 2021. In addition, zymography measurements were performed as previously described [47].

### 2.4. Assessment of Viral RNA Load with Quantitative PCR with Reverse Transcription (RT-qPCR)

RNA extraction from patient plasma was performed using QIAmp(r) Viral RNA Mini Kit (Qiagen) following the manufacturer’s instructions. Two-step reverse transcription using SuperScript II (Invitrogen, Thermo Fisher Scientific, Carlsbad, CA, USA) was used to generate cDNA for PCR by the QuantStudio5 series (Applied Biosystems, Foster City, CA, USA). RT-qPCR reactions were carried out using TaqMan^TM^ Fast Advanced Master Mix (Applied Biosystems, Thermo Fisher Scientific Catalog # 4444965), with SNV infection confirmed and copies calculated using a previously published set of primers based on detection of the S-segment of the SNV genome [48]. Copy number was determined using a plasmid (pFastBac1) containing the SNV N insert (custom generated by Genscript, Piscataway, NJ, USA) as the standard. Human ACTB (Beta Actin) primers with a VIC(tm)/TAMRA(tm)-dye probe (Applied Biosystems, Cat. # 4310881E) were used as an endogenous positive control along with SNV-infected VERO E6 cells (SN77734).

### 2.5. Statistical Analysis

Before statistical analysis, plasma biomarkers’ concentrations were transformed into natural logarithmic values to approximate a normal distribution. Principal component analyses were also performed using GraphPad Prism, Version 9.2.0 (https://www.graphpad.com/scientific-software/prism/ accessed on 9 August 2021) or R. Unsupervised hierarchical cluster analyses were performed using R package heatmap 3 [49] with Euclidean distance as the dendrogram branch length. Pearson correlation coefficients were derived from the study of serial samples for each patient to visualize clustering between cytokines, active uPA, total uPA, and PAI-1 measured in individual patient serial samples.

## 3. Results

### 3.1. Study Group

Our study cohort comprised 13 patients with confirmed HCPS diagnosis [21] admitted to the University of New Mexico Hospital (UNMH) and enrolled in a study between 2006 and 2012. We measured levels of plasma cytokines and chemokines and leukocyte profiles (from retrospective clinical data). In addition, we stratified the patients into mild (Class I), severe based on the need for ECMO and recovery (Class II), and non-survivors (Class III). Patients who developed moderate or severe disease did not differ significantly concerning age or sex; however, our sample size did not provide enough statistical power. Class II and Class III patient characteristics, including demographics, illness course, and outcomes, are listed in Table 1.

Because of variable time lapses between initial infection, disease onset, and admission, study cases were at various chronological stages of HCPS. Therefore, the time coordinate on the graphs are normalized to days post admission to UNMH. Thus, in the graphs, day 1 refers to the first sample and bears no temporal relationship to the actual time post infection or symptom onset as described in the notes to Table 1. In addition, three cases were possibly afflicted with bacterial coinfections (260, 306, and 319), which might have confounded the HCPS related etiology, as will be noted throughout the manuscript.

### 3.2. Overt Neutrophilia Is Associated with HCPS Severity and Mortality

**Figure 1:**
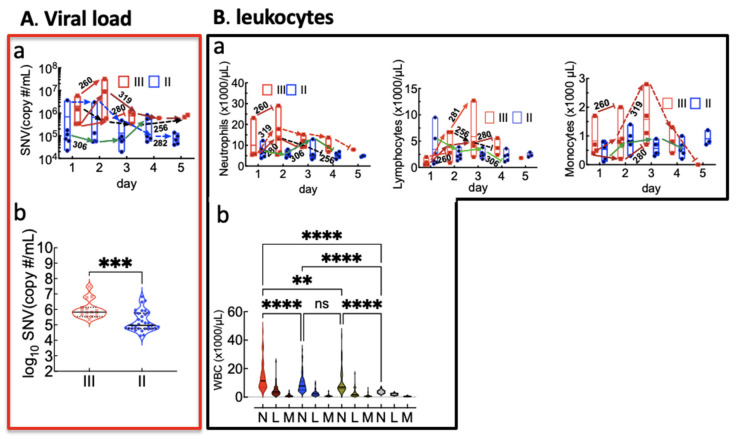
**A** (**a**) Top panel shows viral load in Class II and Class III patients measured in longitudinal plasma samples. Arrows are used to show the trajectory of select cases, that are discussed in the manuscript (**b**) The bottom panel shows a comparison of grouped mean viral load data of Class III and Class II cases using a paired *t*-test; *** *p* < 0.001; violin plots; solid lines, median; dotted lines, quartiles. **B** (**a**) The panel shows longitudinal expression of white blood cells (N), neutrophils (N), lymphocytes (L), and monocytes (M) for Class II (blue) and Class III (red) measured in this study. (**b**) The bottom panel shows retrospective chart review data of N, L, and M count, extracted from 15 Class I, 22 Class II, and 15 Class III HCPS cases. Each case comprised WBC data collected during the patient’s length of stay at UNMH. Levels of N, L, and M were compared by ordinary one-way ANOVA with Tukey’s correction for multiple comparison tests; ** *p* < 0.01, **** *p* < 0.0001. Only Class III N counts were significantly different from Class II, I, and controls.

Figure 1A(a) shows the respective viral loads of Class II and Class III cases, where the mean viral load in Class III cases was significantly higher than Class II Figure 1A(b). The results are consistent with previous studies showing a positive correlation between disease severity and patients with a high viral load [50,51].

In Figure 1B(a), the top panel shows longitudinal changes in leukocytes as a result of infection. Two Class III cases (260 and 319) with the highest peak levels of the viral load presented significant elevation in neutrophils (N) and monocytes (M), indicating a robust innate immune response to infection [52]. Due to the small sample size of the study population, we also analyzed the longitudinal complete blood counts with differentials (CBC with diff) of N, lymphocytes (L), and M from a retrospective review of medical records of 52 HCPS patients (15, 22, and 15 Class I, II, and III cases, respectively). Interestingly, over the length of stay of case patients at UNMH, only Class III patients’ neutrophil counts were significantly elevated compared to other categories, including controls (Figure 1B(b)).

### 3.3. Peak Innate and Adaptive Immune Responses in Class III Cases Are Higher Than in Class II Cases

Table S1 summarizes the measured medians and concentration ranges of the target cytokines: IL-15, IL-18, IL-12p40, IL-1α, TNF-α, IFN-γ, IL-1β, GM-CSF, IL-22, IL-6, IL-4, IL-13, IL-25, IL-2, IL-10, TGF-β1, MCP-1/CCL2, FLT3-L, IL-21, G-CSF, IFN-α, and IFN-β. We did not detect seven cytokines, IL-12p40, IL-1β, GM-CSF, IL-13, IL-25, and IFN-β, in most patients except in some Class III patients. Notably, IL-12p40 was solely detected in patient 260 plasma samples at the extreme levels of 10,906.8 and 11,260.6 pg/mL on days 1 and 2. Overall several cytokines of interest were below the detection limit in control samples. In addition, differences between cytokine expression in Class I (n = 2) and controls (n = 5) were marginal. Thus, our analysis was focused on comparisons between Class II and Class III.

**Figure 2:**
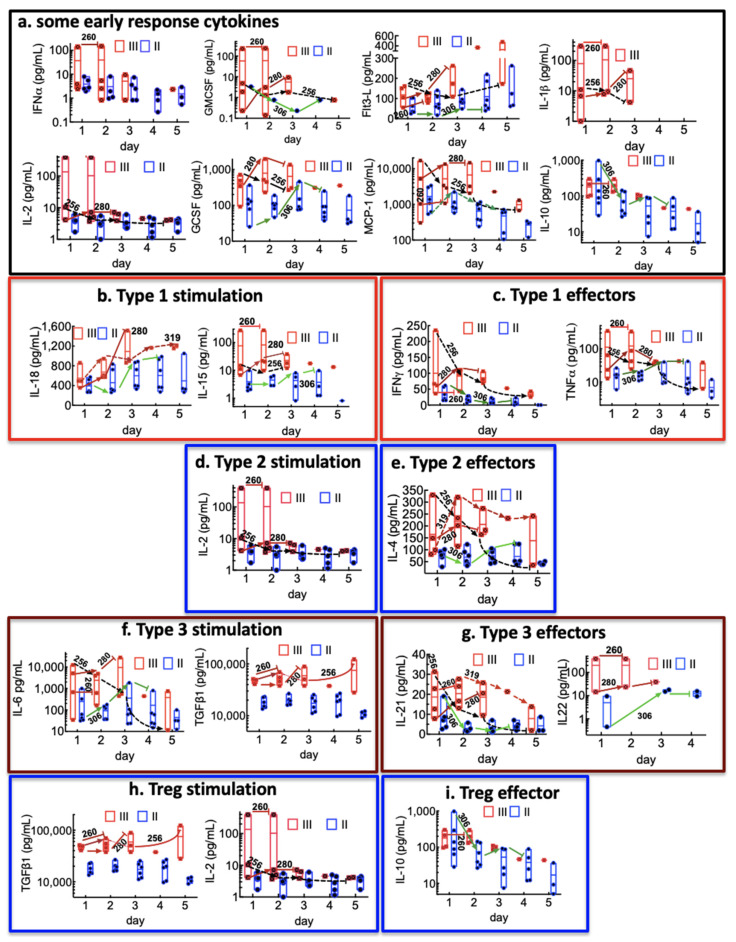
Overview of immunological features in HCPS patients in Class II (ECMO survivors) and Class III (terminal) patients. Arrows and the patient’s ID number indicate the longitudinal expression of the leukocytes for specific patients. (**a**) Plots of cytokines associated with early innate immune responses. (**b**,**c**) Cytokines associated with type 1 immunity. (**d**,**e**) Cytokines associated with type 2 immune response; (**f**,**g**) Type 3 immunity; (**h**,**i**) Treg differentiation.

Innate immune cells, comprising neutrophils, macrophages, monocytes, dendritic cells, and natural killer cells, function as sensors of infection. In general, it is believed that the magnitude of the effector response to infection is governed by how the immune system calibrates pathogen invasiveness, viability, replication, and virulence activities [52]. The stimulating cytokines measured herein included type 1 interferons (IFN-α/β), IL-6, IL-1β, IL-1α, IL-15, IL-18, TGF-β1, TNF-α, FLT3-L, GM-CSF, IL-2, IL-10, G-CSF, MCP-1, and IL-12p40 for case 260 (III) only. It is presumed that the stimulating cytokines acted on a variety of lymphocyte populations, including innate lymphoid cells (ILCs), innate-like lymphocytes (ILLs; including natural killer cells, mucosal-associated invariant T (MAIT)), and epithelial gamma delta T cells, follicular helper T cells (CD4+ T cells), and tissue-resident memory (TRM) cells [52]. The data show that the peak expressions of innate immune mediators IFN-α, the inflammasome-linked IL-1 family cytokines (IL-1β, IL-18), and TNF-α, FLt3-L, G-CSF, GM-CSF, and MCP-1 were substantially elevated in Class III compared to Class II cases (Figure 2a). We organized the results regarding their differentiation from naive CD4+T cells to T helper (Th1, Th2, Th17, and Treg) lineages [53]. The peak expression of Class III, Th 1 or type 1 stimulating cytokines (IL-15, IL-18, and IL-12p40 detected for 260 only), Th2 or type 2 (IL-1α), and Th17 or type 3 (IL-6, TGF-β1, and (IL-1α, IL-1β)) was much higher compared to Class II, as were the type 1 (IFN-γ), type 2 (IL-4), and type 3 (IL-21, IL-22) effector cytokines (Figure 2b–g). For regulatory T cells (Treg), the stimulating cytokines, TGF-β1 was significantly higher (≥ 2 fold) on all days for Class III than Class II. Case 260 was notable for the expression levels of several cytokines that were ≥10 times higher than all the cases. Interestingly, the Treg effector cytokine IL-10 was notably three-fold higher for Case 306 on day 1 than all the cases of the cohort. However, IL-10 steeply declined on subsequent days. As noted in Table 1, Case 306, with a putative bacterial co-infection (Table 1), presented an asynchronous immune response. On day 1, the patient’s lymphocytes (Figure 1B(a), GM-CSF, IFN-γ, IL-21, and IL-10, were at the post-peak declining phase. In contrast, increasing expression of SNV (Figure 1A(a), FLT3-L, G-CSF, IL-18, IL-15, IL-4, IL-6, and IL-22 peaked on day 3 or 4. These successive waves of the immune response suggest a two-tiered response to different pathogens [52].

**Figure 3:**
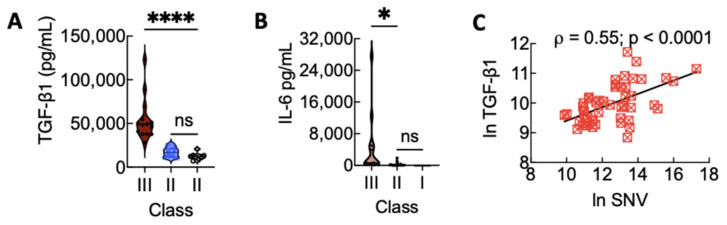
(**A**) Relative expression of TGF-β1 in Class III, II, and I. (**B**) Relative expression of IL-6 in Class III, II, and I on all days. Levels of active TGF-β1 and IL-6 were compared by ordinary one-way ANOVA; **** *p* < 0.0001, * *p* < 0.05. (**C**) The association between TGF-β1 and SNV during HCPS assessed by Pearson’s correlation test showing a positive correlation between the variables. The figure was produced in GraphPad Prism version 9.2.0.

At this juncture, it is worth noting that the mechanism of CD4+ T cells differentiating into type 3 or Treg cells is linked to the same TGF-β1-dependent signaling mechanism. However, this outcome is regulated by proinflammatory cytokines present during the activation of naive CD4+ T cells [53]. Proinflammatory IL-6 and IL-21 drive T-cell differentiation toward type 3 when TGF-β1 is present at a low concentration [54]. TGF-β1 can induce Tregs in the presence of IL-2 [55]. With this in mind, our data suggest that, in general, the high co-expression of IL-6 and TGF-β1 in Class III cases favored type 3 immunity. Class II cases expressed significantly low levels of IL-6 in which is expected to promote Treg differentiation (Figure 3A,B).

Case 260 (III) presented several anomalous results, notably expression of extremely high levels of IL-12, as noted above, and ≥ 10-fold levels of GM-CSF relative to other cases in the cohort (Figure 2a). In addition, other inflammatory cytokines, such as (IFN-α/β), IL-1β, IL-1α, IL-15, TNF-α, IL-2, and IL-22, were highly expressed. The patient’s immediate past medical history indicated a urinary tract infection (UTI) at the time, overlapping with her symptom onset and an eventual positive diagnosis for HCPS. It has been suggested [52] that the innate immune response comprises an evolutionarily, fine-tuned mechanism of sensing and identifying specific pathogens and setting a critical choice of an appropriate response. We suggest that the patient’s expression and magnitude of IL-12 and GM-CSF are consistent with an immune system’s CD8+ T cells and type 1-mediated defense against intracellular bacteria and protozoa [52] juxtaposed with emergent SNV-related pathophysiology.

Another point of interest involves the difference in the expression levels of TGF-β1 for Class III and Class II, which was continuously significant (≥2) for all days (Figure 2f). Because previous studies have shown that sustained elevation of TGF-β1 downregulates type 1 interferon antiviral response in mice [56], we asked if there was a positive correlation between the expression of TGF-β1 and SNV viral load. As shown in Figure 3C, we found a positive and significant correlation between the two variables. Type 1 interferons IFN (α/β) are the earliest innate immune response cytokines to infection [52]. Thus, the positive correlation between TGF-β1 expression and viral load during HCPS suggests the possibility of TGF-β1 recapitulating a pro-viral posture as previously reported [56].

### 3.4. Plasma Levels of uPA and PAI-1 and Their Link to Lymphocyte and Cytokine Expression

**Figure 4:**
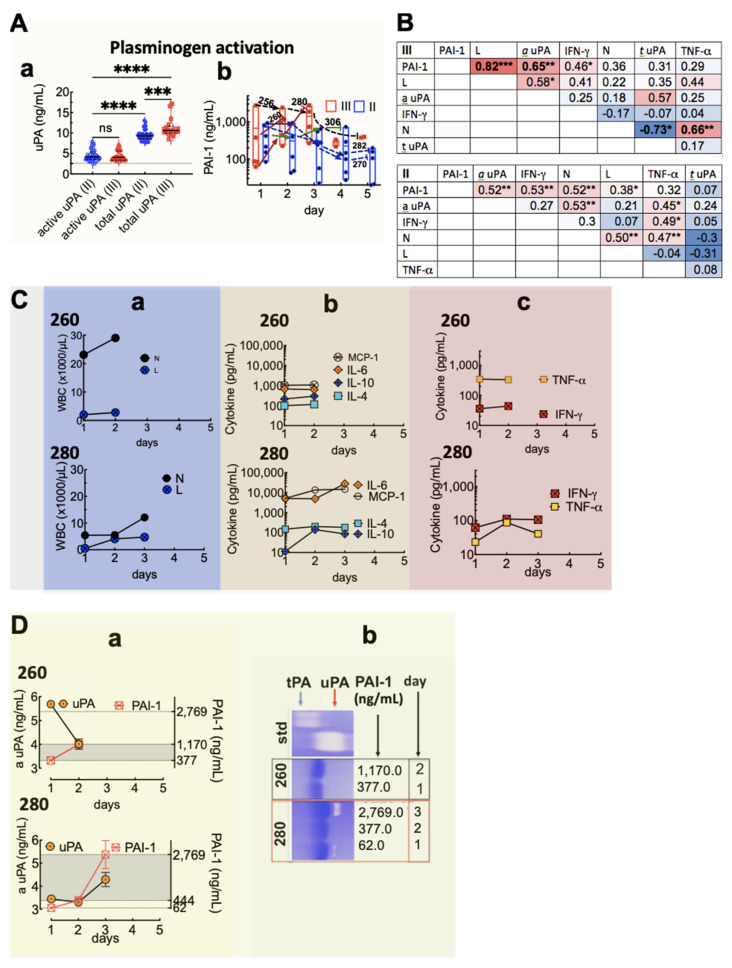
Peak plasma levels of uPA and PAI-1 are linked to lymphocyte expansion and IFN-γ expression. (**A**) Plasminogen activation. (**a**) Data plots of active and total uPA for Class II and III cases. Data were measured for up to 5 days for each case depending on sample availability; see Figure S1 for changes over time. (**b**) Longitudinal data plot of active PAI-1 expression for Class II and III cases. (**B**) Pearson’s correlation matrices compared PAI-1, active uPA (a uPA), total uPA (t uPA), lymphocytes (L), neutrophils (N), and IFN-γ measured in Class III and II patients. Correlations are classified according to the following scale: 0–0.19 very weak, 0.2–0.39 weak, 0.4–0.59 moderate, 0.6–0.79 strong, 0.8–1.0 very strong. Levels of active uPA and total uPA were compared by ordinary one-way ANOVA with Tukey’s correction for multiple comparison tests; * *p* < 0.05, ** *p* < 0.01, *** *p* < 0.001, **** *p* < 0.0001 (**C**) Neutrophilic- (Class III patient 260) and lymphocytosis (Class III patient 280) mediated immune response drives differential plasminogen activation responses. (**a**) Comparison of N and L expression for 260 and 280. (**b**). Expression of IL-4, IL-10, IL-6, and MCP-1. (**c**) Expression of type 1 effector cytokines. (**D**) (**a**) ELISA measurement of a uPA and PAI-1. (**b**). Zymography measurement of tPA, uPA, and ELISA measurement of PAI-1.

Our previous studies showed that excessive PAI-1 upregulation is linked to end-stage HCPS [37]. uPA is a chemotactic factor for leukocytes and a lymphocyte mitogen [57]. Net plasminogen activity is regulated by the balanced expression of urokinase-type plasminogen activator (uPA) and plasminogen activator inhibitor type 1 (PAI-1). Therefore, we asked whether excessive PAI-1 upregulation was positively correlated to lymphocyte expansion and its primary effector cytokine, IFN-γ. We used ELISA assays for uPA and PAI-1 to determine the relative balance between leukocyte expression, secreted cytokines, uPA, and PAI-1 proteins. The plasma concentration of total uPA in healthy patients is 1–2.5 ng/mL [58]. In Class II patients, the median values of active and total uPA (inactive and PAI-1 complexed to uPA) measured in longitudinal samples were 4.1 (range: 2.5–7.9) ng/mL and 9.3 (range: 7.7–13.1) ng/mL, respectively (Figure 4A(a); see Figure S1 for changes over time). For Class III cases, the median expression of active and total uPA was 4.1 (range 3.1–7.1) ng/mL and 10.6 (range: 8.4–17.1) ng/mL, respectively (Figure 4A(a)). The plasma concentration of PAI-1 ranges between 6 and 80 ng/mL in healthy controls [59,60]. For Class II, peak levels of PAI-1 varied from 151 ng/mL to 938 ng/mL compared to 738 ng/mL and 2770 ng/mL for Class III (Figure 4A(b)). Excessive uPA activity is known to drive PAI-1 upregulation. Yet, our data showed no significant difference in uPA activity between Class II and III, where attendant PAI-1 expression was significantly elevated in Class III.

Others have shown that IFN-γ and TNF-α differentially modulate the activity of urokinase expression in mononuclear phagocytes [42,61]. Therefore, we hypothesized that the efficacy of PAI-1’s activity against uPA was dependent on the cytokine profile measured in the patient’s plasma. Thus, to draw integral comparisons of L and N’s relative effects on plasminogen activation, we assessed pairwise Pearson’s correlations of IFN-γ, TNF-α, uPA, and PAI-1 measured in Class III and II samples (Figure 4B, Supplementary Figure S2). For Class III, we detected a strong and significant positive correlation between PAI-1, L, and active uPA (*a* uPA, and IFN-γ). However, TNF-α was strongly correlated to N only. Class II PAI-1 was moderately but significantly correlated to *a* uPA, IFN-γ, and N and weakly correlated to L. Additionally, L and N were correlated, as was IFN-γ and TNF-α. Taken together, the data suggest that L and IFN-γ primarily drive Class III plasminogen activity. In contrast, in Class II, both L and N contribute to plasminogen activation.

We next compared cytokine-expression profiles of two Class III patients. The first was case 260, a patient with a severe immune response with overt signs of neutrophilic leukocytosis and expressing lymphocytes in the normal range (260, Figure 4C(a)); the second was case 280, presenting an acute expansion of lymphocytes and ≥ 2-fold levels of PAI-1 compared to 260 (280, Figure 4C(a)). The expression of IL-10 and IL-4 was comparable for both cases (Figure 4C(b)). However, significant differences existed in the levels of IL-6 (40-fold higher) and MCP-1 (15-fold higher) in Patient 280 relative to 260 (Figure 4C(b)). The significantly higher concentrations of IL-6 and MCP-1 suggested that the transition from innate immune response to adaptive immunity [62,63,64] was substantially more robust in Patient 280. For Patient 280, IFN-γ was three times higher than Patient 260. However, TNF-α was >2-fold higher in Patient 260 than its peak level in Patient 280.

To determine the effect of the differential expression of TNF-α and IFN-γ in the two patients, we compared the expression of active uPA relative to PAI-1 (260 (III) and 280 (III) using ELISA (Figure 4D(a)) and zymography (Figure 4D(b)). For Patient 260, the concentration of active uPA was sensitive to PAI-1 upregulation, as indicated by a diminution in uPA activity with increasing PAI-1 expression from 377.0 to 1170 ng/mL. In contrast, for Patient 280, active uPA decreased with the initial increase in PAI-1 from 62 to 444 ng/mL but subsequently upturned despite a seven-fold increase to 2770.0 ng/mL PAI-1 expression the next day. Interestingly, the overall concentration of active uPA in Patient 280 was lower than that secreted by Patient 260. Zymography measurements found no measurable uPA activity in Patient 260, indicating efficient inhibition of uPA by PAI-1. Concerning Patient 280, low-level uPA activity was detected on day 1, when PAI-1 expression was in the normal range. On day 2, an increase in PAI-1 secretion inhibited uPA activity. However, on day 3, robust uPA activity was evident on the zymogram despite the peak elevation of PAI-1. The data broadly suggest that PAI-1 activity towards uPA is impaired in Patient 280 compared to 260. We suggest that the IFN-γ replete immune environment of Patient 280 is likely to be driven by pro-uPA/uPAR plasminogen activation pathway, which binds to PAI-1 with five-fold less affinity and is reversible, permitting persistent uPA activity [40,46].

### 3.5. Longitudinal Immune Profiling of Cytokine and Plasminogen Activation Factors in HCPS

**Figure 5:**
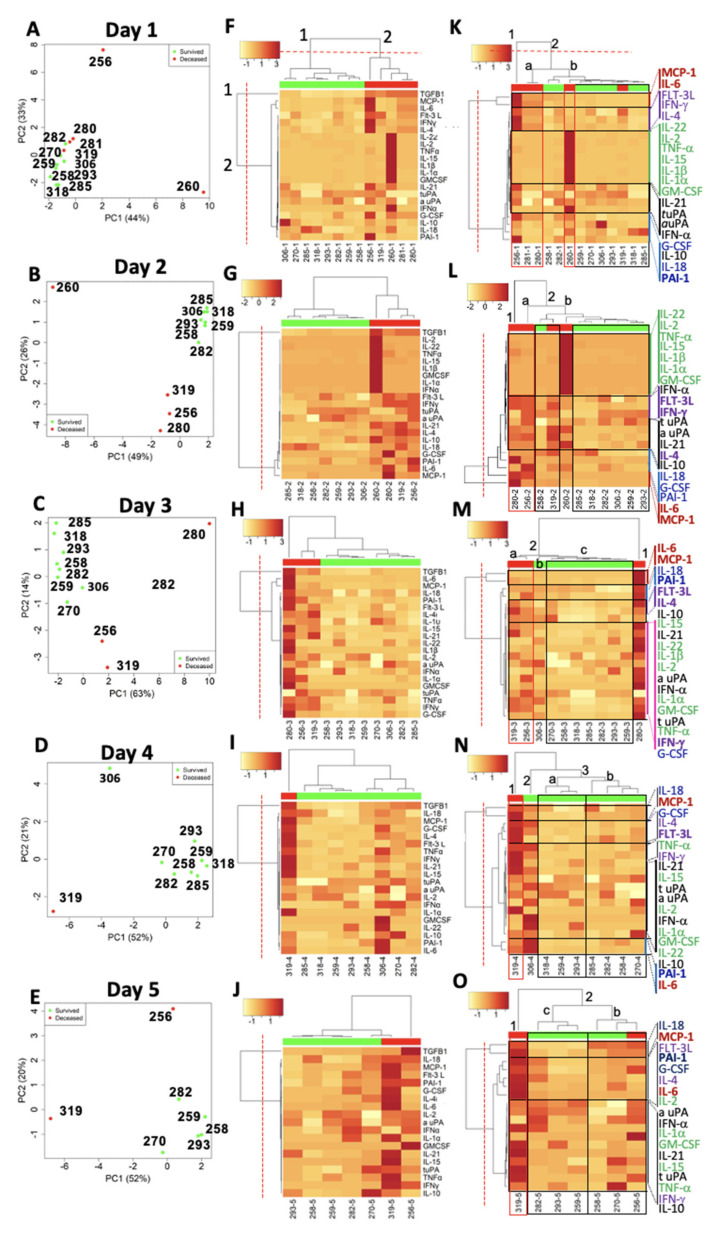
Differential expression of inflammatory mediators in longitudinal plasma samples of HCPS distinguish between survivors and non-survivors. (**A**–**E**) Principal component analysis (PCA) of inflammatory mediators 15 cytokines, a uPA, t uPA, PAI-1 N, L, and M in 2 Class I (318 and 285), 6 Class II patients (258, 259, 270, 282, 293, and 306), and 5 Class III patients (256, 260, 280, 281, and 319). Each panel corresponds to days (day 1–5) of sample collection. (**F**–**J**) Heat map cluster analysis-based Euclidean distance similarity between inflammatory mediators of survivors (green horizontal bar) and non-survivors (red horizontal bar). Inflammatory mediator clusters (rows) are listed on the right edge of the heatmap, and patient clusters (columns) are indicated on the top edge. The colors represent the log-concentration range. (**K**–**O**) Heat map cluster analysis recapitulated with TGF-β1 excluded.

To further evaluate potential drivers of severe HCPS outcomes in an unbiased manner, we performed an unsupervised clustering analysis of cytokines and plasminogen activation that included all 13 patients and all timepoints (Figure 5). PCA plots convert an N-dimensional matrix of correlated variables into a 2-D graph comprising principal components: PCA1 being the *x*-axis that spans the most significant variation in expression among the test variables (cytokines, PAI-1, etc.) versus PCA2 axis spanning the second most significant variation expression of test variables. The longitudinal data for some Class III patients was limited due to patient death (260 and 280) or limited sample availability (256, 281), as indicated by changes in the number of Class III patients on the daily PCA plots (Figure 5A–E). The survivor cases were clustered near the origin of PC1 and PC2 axes over the five days except for Patient 306 that segregated from the survivor cluster on day 4 (Figure 5C,D) due to an upregulation of inflammatory mediators (306 in Figure 2). The distribution of the survivors near the origin throughout the time course suggested that their immune profile changed minimally relative to Class III patients. On day 1, three of five Class III patients with a dynamic-phase immune response (280, 281, and 319) were distributed among the survivors, suggesting that the latter shared common markers with dynamic-stage Class III patients. Patient 260’s coordinates along the PC1 axis indicated that the most significant immunological response was linked to 260.

In contrast, Patient 256’s appearance along the PC2 axis indicated that Patient 256’s late-phase immunological profile was greatly different from 260. Patient 280’s immune response reached an apex on day 3 along PC1 as Patient 256’s immune response declined. Patient 306 was segregated from the survivor cohort along the PC2 axis on day 4, suggesting that drivers of 306’s immunity were orthogonal to 319.

For heat map visualization of the PCA, we used agglomerative hierarchical clustering of the Euclidean distance patterns of the 19 PCA analyzed variables (16 cytokines, uPA, tPA, and PAI-1) to identify distinct cluster boundaries for the patient (horizontal axis) and cytokine/plasminogen activation (vertical axis) dendrograms (Figure 5F–J). Two clusters defined the segregation of Class III and the survivors (Class I and II). In addition, the intra-cluster Euclidian distances among the survivors in Cluster 1 were short, suggesting strongly correlated immunological states of the cluster members.

In contrast, the intra-cluster distances among Class III patients in Cluster 2 were longer, reflecting the heterogeneous distribution of highly upregulated cytokines displayed along the vertical axis. The inflammatory mediators were divided into 2 clusters, with Cluster 1 containing TGF-β1 only and Cluster 2 containing the rest of the inflammatory mediators. TGF-β1 was expressed at significantly higher concentrations in all Class III patients relative to Class I and II, as manifested by the magnitude of the associated Euclidian distance separating TGF-β1 from the other inflammatory mediators along the vertical axis.

To obtain a granular view of the cytokine cluster boundaries, we excluded TGF-β1 from the cluster analysis (Figure 5K–O). The exclusion of TGF-β1 did not change the cluster distribution of cytokines. However, the reorganization of patient clusters was Class III patients, 319 and 260, co-mingled with the survivors. Thus, the distribution of patients into different clusters was dependent on the magnitude of specific cytokine concentrations, such as MCP-1 and IL-6, notably for 256 and 280, with available samples for ≥3 days.

Correlation between MCP-1 and IL-6 (Cluster 1) emerged and persisted for 3 days in the two Class III patients, suggesting a robust transition from innate to adaptive immune response [62,63,64]. Patient 260 was characterized by peak expression of cytokines in Cluster 1 (IL-22, IL-2, TNF-α, IL-15, IL-1β, IL-1α, and GM-CSF), on days 1 and 2 before the patient died. In this patient, TNF-α and IL-1α/β expressed at significantly high levels of all Class III cases are functionally linked by their propensity to activate the NF-κB pathway responsible for the transcriptional induction of the inflammatory mediators [65].

Patient 319 emerged from the survivor grouping on days 4 and 5 due to most Class III patients’ deaths and correlated rise of IL-18 and MCP-1, consistent with the known activity of IL-18 as an inducer of MCP-1 expression [66]. However, the peak cytokine concentrations of 319 were two orders of magnitude lower than peak concentrations of 256 and 280, indicating less severe inflammation associated with 319.

### 3.6. Robust Adaptive Immune Profile Is Correlated to Severity

**Figure 6:**
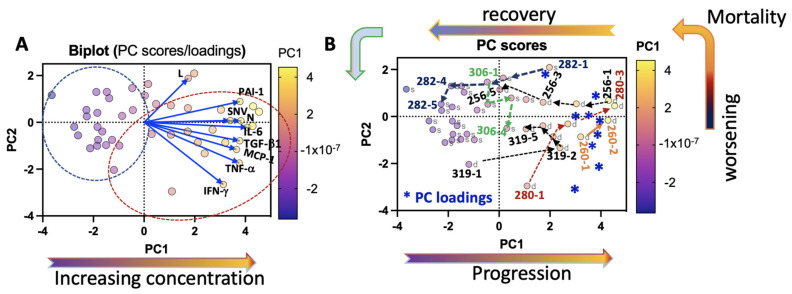
Principal Component Analysis (PCA) of patient variables, IFN-γ, IL-6, MCP-1, TNF-α, PAI-1, TGF-β1, SNV, N, and L, distinguishes survivors and decedents. (**A**) Biplot of PC scores graph and loadings. The PC scores data points are color-coded based on their magnitude on the PCA1 color scale. The loadings data are attached to the blue vectors and represent patient variables. The magnitude and direction of the vectors represent the correlation between the loadings and the PC scores. The angle between two vectors is proportional to the degree of correlation for different vectors, with smallest angles meaning high correlation, orthogonal (90°) as uncorrelated, and opposing (180°) as negatively correlated. (**B**) PC scores plot identifies survivors (s) and decedents (d). Class III cases are displayed as case numbers on different days. Dotted lines connect longitudinal data points and show a directional change in immune response and correlated loading variables (* displayed as vectors in Figure 6A). The original Biplot and PC scores figures were produced in GraphPad Prism version 9.2.0.

Severe HCPS is primarily driven by dysregulated inflammation; for our model, we selected the subset of variables: IL-6, MCP-1, TGF-β1, IFN-γ, TNF-α, viral load, PAI-1, lymphocytes (L), and neutrophils (N), which were measured at high levels in our study cohort. We used unsupervised clustering analysis based on Principal Component Analysis (PCA) to find distinct clustering patterns of survivors and decedents (Figure 6).

The results are shown as a distance biplot of PC scores (longitudinal patient samples) and PC loadings vectors (immunological variable) (Figure 6A). The PC scores represent the proportionate contribution of each immunological variable (e.g., L, PAI-1, SNV, IL-6, etc.) on each day for each patient on a PC1/PC2 plot. The magnitude and direction of the PC loadings vectors indicate a proportionate concentration of the immunological variables and correlation with longitudinal case samples. As shown on the color scale (Figure 6A), PC1 has a robust positive presentation for SNV, TNF-α, IFN-γ, IL-6, MCP-1, PAI-1, N, and moderate L. In the Biplot, the PC loadings vectors appear on the right-hand side. The longitudinal Class III case samples are localized, indicating that the high concentration inflammatory mediators and principal components are positively correlated. The correlations between the immunological variables are related to the angles between the vectors that they represent. Therefore, when two vectors form a slight angle, the two variables they represent are strongly correlated, e.g., SNV and N (Figure 6A). This result is consistent with the data shown in Figure 1, which indicates that cases 260 and 319 have the highest corresponding values of N and SNV. Conversely, vectors appearing at ~180° are negatively correlated, and those that seem nearly orthogonal (90°) are likely not correlated.

Figure 6B shows an annotated PC scores graph displaying longitudinal 2-D coordinate-changes for each case under the influence of the inflammatory mediators. We used asterisks to represent the location of PC loadings vectors in Figure 6B to conserve figure clarity. The PC1 axis represents increasing immune dysregulation as represented by the magnitude and direction of the PC loadings vectors. PC2 axis represents case correlations with different mediators.

Arrows and case ID indicate the immune response-correlated lateral movement of case subjects on different days. As indicated by the arrows, the longitudinal trajectory of Case 319 illustrates how increasing inflammation (cf. Figure 2) is associated with the significant lateral movement by 319 (∆PC1; 3 logs) towards the PC loadings vectors on day 2. However, the subsequent vertical translations (PC2 axis) were relatively small and retrogressive, indicating a diminution of inflammation in this patient. This result is consistent with the clinical findings; that 319 died from complications due to ECMO. In contrast, Patient 280’s rapid migration primarily along the PC2 axis (∆PC2; 3 logs) on day 2 was followed by a translation along PC1 and PC2 ordinates. This result indicates that 280 experienced a rapid and correlated increase in all the proinflammatory immunological variables used in the analysis. The data analysis suggests that patients with the most elevated proinflammatory state localized in the top right quadrant. Patients such as 256 (age 69, BMI 27.1), 280 (age 30, BMI 32), and 282 (age 16, BMI 23) were localized in the top right quadrant on day 1. However, their respective coordinates along the PC1 axis and raw data indicated that 256’s and 280’s immunological variables were more significant than 282’s. Case 280 died on day 3 upon reaching the top right quadrant. On their respective timeline, 256 and 282 rapidly moved from top right to the left on the PC scores distance graph away from the immunological variables, where 256 died on day 5. Case 282’s deadly inflammatory condition rapidly improved, having traversed a significant abscissa space (∆PC1; >4logs) of the PC distance graph to co-localize with other Class II cases. However, the patient’s illness course lasted 30 days (Table 1). Although our results show a positive association between mortality and overweight/older age or obesity, we are not aware of a statistically significant study of such a correlation; thus, this observation cannot be generalized.

As previously noted in Section 3.3, Class II Case 306 presented an asynchronous immune response, which was highlighted by the declining peak expression of IL-10, lymphocyte counts (L), IFN-γ, and IL-21 and increasing N, M, IL-18, IL-15, TNF-α, IL-6, and IL-22 within the same time interval. In Figure 6B, good correlations with the PC loadings vectors determined the trajectory of 306’s coordinates. On the graph, 306-1 to 306-2 marked the decline of the first wave of bacteria-induced inflammatory mediators. In this case, the PC loadings vector *L* was correlated to 306-1. Localization of 306-3 was likely influenced by the second wave of SNV-initiated *PC loadings vectors*; N and IL-6 were the main drivers of the patient’s pro-inflammatory state on day 3. The ordinate movement due to 306-4 was likely correlated with TNF-α. Thus, we suggest that the second wave of inflammatory mediators was more critical than the first because on day 1, 306-1 was localized among near-convalescing Class II cases and ended in the Class III zone. Given that the entire illness course for this patient lasted 35 days before being discharged to a skilled nursing inpatient facility indicates that 306’s condition remained severe long after the time frame of this study.

### 3.7. Differential Development of Innate and Adaptive Immunity Separates Survivors and Non-Survivors of HCPS

An effective host immune response is generally the outcome of both pro-and anti-inflammatory elements modulated to eliminate the pathogen with minimum injury to the host. Anti-inflammatory regulatory T-cells (Tregs)-mediated suppression of proinflammatory responses often plays a protective role against immunopathology [53]. The balance is not necessarily stoichiometric, but it is qualitative coordination in downstream activation and inhibition [67]. Proinflammatory cytokines govern the TGF-β1-mediated differentiation balance between Th17 cells and Tregs [68]. In the presence of TGF-β1, IL-6 induces Th17 differentiation from naïve CD4+ T cells, whereas IL-6 inhibits TGF-β1-induced Treg development. Tregs are the product of TGF-β1 signaling in the presence of IL-2 [55]. Treg cells secrete anti-inflammatory cytokines IL-10 and TGF-β1 to suppress the activity of various immune cells.

We next analyzed the spatial distribution of longitudinal Class II and III PC components relative to IL-2, IL-4, IL-6, IL-10, and TGF-β1 vectors in PC Biplot distance graphs. Our goal was to determine whether Class II PC components were more likely to be correlated to IL-2, TGF-β1, and IL-10 than Class III cases.

**Figure 7:**
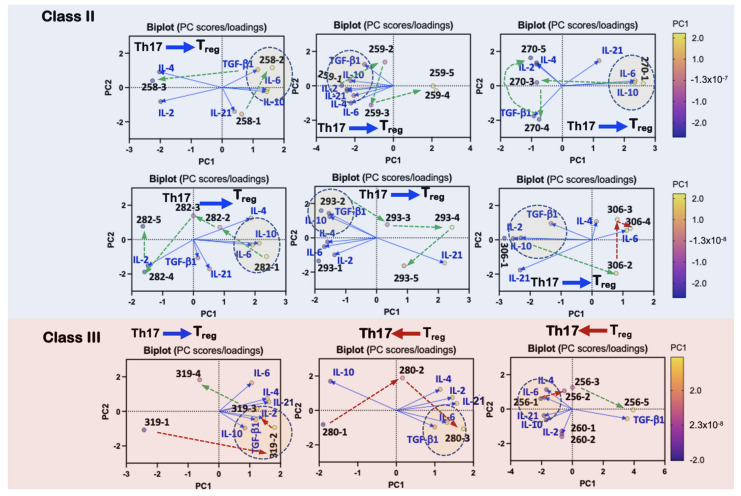
Immune correlates of Treg-mediated outcomes. Principal Component Analysis (PCA) of Class II and III patient variables IL-2, IL-4, IL-6, IL-10, and TGF-β1 associated with Treg differentiation. The Th17/Treg blue or red arrows indicate the favored choice of differentiation. Patient PC scores are shown as case ID and days post admission (e.g., 258-1). Conditions that favor Treg differentiation are indicated by the dotted oval highlighting the correlation of Treg promoting cytokines, e.g., correlated clusters of IL-10 (effector Treg cytokine), IL-6 (low expression), and TGF-β1. For Class III, 280 demonstrates unchecked growth of a proinflammatory state based on the spatial positioning of 280-1, 280-2, and 280-3 relative to the cytokines. The 180° separation of the low-concentration IL-10 and the closely correlated TGF-β1/IL-6 signaling axis is consistent with prevailing conditions for an imbalance between Th17/Treg states in favor of Th17. 260-1, 260-2: insufficient data for only two days.

Based on the above criteria for effective anti-inflammatory activity, the longitudinal profile for Treg potential was strong for Class II patients (Figure 7). The correlation between Class II cases and inflammatory mediators indicated movement away from the proinflammatory IL-6/IL-21 vectors towards pro-Treg TGF-β1/IL-2/IL-10-correlated states. For Case 306 on day 1 (306-1), the declining initial wave of bacteria-induced inflammation was limited by a strongly correlated TGF-β1/IL-2/IL-10 signaling checkpoint, which likely tempered the second wave of the proinflammatory state represented by the close correlation between IL-6 and 306-3 and 306-4 (Figure 7). Case 319 demonstrated a robust Treg phenotype at 319-2’s proximity correlation to TGF-β1/IL-2/IL-10. This result is consistent with the arrested development of the proinflammatory state, as illustrated by 319’s trajectory as rendered in Figure 6.

**Figure 8:**
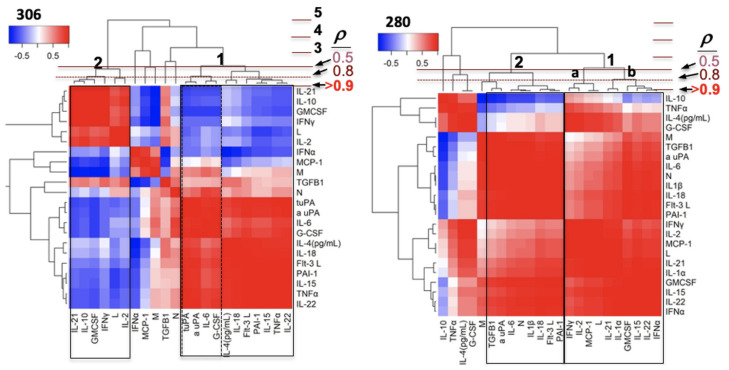
Unsupervised comparisons of cytokines measured at days 1–3 timepoints in each patient. Heatmap and cluster analysis of inflammatory mediators (cytokines, chemokines, growth factors, leukocytes, coagulation factors) measured in clinical patient plasma samples. A horizontal line was drawn in each heat map to indicate a suggested separation of inflammatory response patterns based on the cluster dendrograms and visual inspection of the heatmaps. The heat maps show pairwise Pearson’s correlations (color-coded: red, positive; blue, negative correlation) among inflammatory mediators sorted along both axes to emphasize object clusters using hierarchical clustering.

To further evaluate the potential drivers for immune dysregulation, we performed an unsupervised cluster analysis of Pearson’s correlations of all patient variables at all time points. We compared Class II Patient 306 and Class III Patient 280. We used the Pearson cophenetic correlation statistic using horizontal lines to denote Pearson coefficients (Figure 8). The heights of merged branches indicate the clusters’ mutual proximal distance, with Pearson’s correlation coefficient as the distance metric. The heat map for 306 presented a two-cluster solution of strongly correlated (*p* ≥ 0.8) cytokines within each cluster. Interestingly, Cluster 1 comprised the second wave of increasing inflammatory mediators, as presented in Figure 2 and Figure 4A: *t* uPA, *a* uPA, IL-6, G-CSF, IL-4, IL-18, FLT3L, PAI-1, IL-15, TNF-α, and IL-22. Cluster 2 contained the first wave of declining cytokines IL-21, IL-10, GM-CSF, IFN-γ, L, and IL-2. TGF-β1 was outside the two-cluster grouping. The split distribution of type 1, 2, and Th17/Treg cytokines mostly between Clusters 1 and 2 is consistent with the asynchronous co-infection of Case 306 by bacteria and SNV manifested in Clusters 2 and 1.

In contrast, for Patient 280, Cluster 1, containing IFN-γ, IL-2, MCP-1, L, IL-21, IL-1α, GM-CSF, IL-15, IL-22, and IFN-α, had a cophenetic similarity coefficient of 0.5 with Cluster 2, (PAI-1, FLT-3L, IL-18, 1L-1β, N, IL-6, a uPA, and TGF-β1). In this case, stimulating and effector cytokines for type1 and Th17 mediators are grouped in the same cluster of close correlates that favored a strong proinflammatory state, which diminished the potential for Treg activity. For example, uPA is known to activate latent proinflammatory cytokines TGF-β1 and IL-6, which drive type 3 immunity [69,70,71]; these inflammatory mediators are closely correlated in Cluster 2, while the effectors IL-21 and IL-22 are equally correlated in Cluster 1b. In addition, L, IL-15, and IFN-γ show a close correlation in Cluster 1. As 280 died on day 3, potential decline of 280′s immune response could be modeled by Patient 256 (Supplementary Figure S4), with a similar profile at peak expression of inflammatory mediators (cf. Figure 7 and Figure 8). It is worth noting that cases 256 and 280, with available samples for ≥ 3 days, show the close clustering of cytokines (IL-2, IL-4, IL-6, IL-15, IL-21, GM-CSF, and GCSF) whose receptors signal via a common pathway involving the Janus kinase (JAK) family of tyrosine kinases and the TYK kinase family members [72], which is a central antiviral mechanism of the host immune defense. While our data are limited, the data for 280 and 256 suggest that the immune indicators of patients of similar immune attributes, which we suggest are linked to refractory HCPS, are characterized by the same cluster occupancy of correlated type 1 and Th17, stimulating and effector cytokines. Heat maps for Class II cases (Supplementary Figure S3) are consistent with immune checkpoints, which allow regulation of the proinflammatory state.

## 4. Discussion

Due to the small sample size, the data represent a limited but representative profile of factors that contribute to the severity of HCPS. Various cell types that function as sensors of infection or damage initiate an innate immune response to viral infection. In general, the magnitude of the adaptive immune response to infection is governed by how the immune system calibrates pathogen invasiveness, viability, replication, and virulence activities [52]. Some Class III patients present dysregulated innate and adaptive immune responses, which might show a robust type 1 and Th17 response characterized by enhanced lymphocyte proliferation and attendant high concentrations of IFN-γ and plasminogen activation (e.g., 280, 256, and potentially 281; however, too few longitudinal samples were available). Elevated secretion of IL-6 and MCP-1 reflects a robust adaptive immune response, which is characterized by lymphocyte proliferation and resultant high concentrations of lymphocyte effector cytokine IFN-γ and plasminogen activation.

uPAR is expressed by antigen-presenting cells (APCs), such as dendritic cells (DCs), monocytes, and macrophages [36,73]. Thus, the concomitant expression of uPA and uPAR by T cells and APCs supports the possibility that the PA system participates in T-cell priming. uPA is a chemotactic factor for leukocytes and a lymphocyte mitogen [57]. The expressions of both uPA and its receptor uPAR are amplified during T-cell activation compared with their levels in resting or naive T cells [73,74]. In this study, patients with the highest peak expression of PAI-1 underwent lymphocyte expansion in the dynamic phase and sustained at end-stage. Our research suggests that the robust expression of IFN-γ was correlated to dysregulated PAI-1 upregulation due to PAI-1 refractory pro-uPA/uPAR [40,46]. Consequently, significantly high levels of PAI-1 were detected in 256 and 280 due to the presumptive overexpression of pro-uPA-uPAR shown herein to be refractory PAI-1 inhibition. In contrast, Class II cases expressed relatively low-level IFN-γ, and uPA was responsive to comparably much lower concentrations of PAI-1.

Interactions of the immune system with co-infecting pathogens can alter the ostensible etiology of a disease caused by a single pathogen. A Class III case, Patient 260′s immune response was distinct from 256 and 280, being underscored by neutrophilic leukocytosis, normal range lymphocytes, and secretion of remarkably high levels of innate immune mediators, including cohort-peak TNF-α and mid-high expression of PAI-1. Neutrophilic leukocytosis with a pronounced left shift is a hallmark of severe HCPS. The left shift is due to the discharge of immature neutrophils, also known as granulocytic myeloid-derived suppressor cells (G-MDSCs), which can suppress T-cell-mediated immune responses [2,75,76]. Inflammasome-linked cytokines enhanced Patient 260’s innate immunity. Her levels of IL-1 (IL-1α and IL-1β), IL-2, IL-18, and TNF-α were several-fold higher than other Class III cases. However, PAI-1 secretion by 260 was less than half that of 280 and 256. Patient 260 was diagnosed with a urinary tract infection five days before admission with elevated temperatures (104.3 °C). On admission, with a clinically confirmed HCPS diagnosis and verified by the presence of plasma SNV RNA herein, she was cannulated for veno-arterial intubation in anticipation of ECMO treatment, reserved for refractory HCPS [9]. However, her overall clinical presentation, which included mild thrombocytopenia (platelets 115,000), ultimately never met ECMO threshold, unlike all the other Class II and Class III cases (Table 1). We hypothesize that an unresolved bacterial infection exacerbated her serious condition, as noted in Table 1 and Section 3.3. of the Results. This idea is substantiated by the patient’s expression of extreme levels of IL-12p40 and GM-CSF associated with host defense against intracellular bacteria and protozoa [52]. Additionally, she presented signs of inflammasome activation, which generally involves Toll-like receptor agonists’ engagement, such as lipopolysaccharide or exposure to TNF-α [77,78,79]. In addition, the binding of extracellular ATP to P2X_7_R triggers caspase-1 activation, which induces IL-1 and IL-18 secretion [80,81], consistent with the abnormally upregulated levels in Patient 260. We suggest that the apparent etiology of the bacterial infection, highlighted by the excessive in-tandem levels of IL-12p40 and GM-CSF, was more significant than SNV-induced HCPS in light of the fact that the patient did not meet the criteria for ECMO. Thus, as with COVID-19 [82,83], other underlying conditions could significantly influence the pathophysiology and outcome. In a similar vein, Class II Patient 306 presented a temporally distinct co-infection of bacteria and SNV, which was apparent in two waves of immune activation involving different inflammatory mediators. The Treg-mediated release of elevated IL-10, designed to modulate to the pro-inflammatory response to the declining bacterial infection, impaired the patient’s T-cell-mediated immune response to emergent SNV effectively. Thus, the patient’s outcome included a protracted convalescence in hospital and then discharged to a skilled nursing inpatient facility.

It is also worth noting that the hyperinflammatory response associated with HCPS is reminiscent of cytokine-release syndrome (CRS) in patients experiencing severe immune reactions to sepsis [84] or COVID-19 [85]. Cytokines, such as IL-1α, IL-1β, IL-6, IL-18, IFN-γ, MCP-1, GM-CSF, and TNF-α, are associated with CRS. A recent study has linked the IL-6-mediated signaling axis with MCP-1, IL-8, and IL-10 to the elevation of PAI-1 [86], recently shown to be elevated in COVID-19 patients [87]. However, the peak expression level of PAI-1 associated with HCPS is significantly higher than PAI-1 measured in COVID-19 [87]. The difference is likely due to higher peak levels of IL-6; 27,651.1 pg/mL measured herein (Table S1) and elsewhere [88] compared to COVID-19; ≤250 pg/mL [89]. The relative magnitude of proinflammatory secretion is consistent with the higher overall case mortality rate of HCPS compared to COVID-19. The common features in the pathophysiology of HCPS and other more prevalent diseases present opportunities for exploring plausible targets for therapeutic targets. Concerning therapeutics development, it is worth noting that Treg-expansion therapy has been explored to treat COVID-19 patients [90].

In summary, patients with HCPS are hospitalized with an average of 3 to 8 days of signs and symptoms. By this time, our data suggest that Class II patients present a regulated inflammatory response associated with recovery. In contrast, Class III is related to ongoing host injury or life-threatening damage. We showed host-response patterns could be conveyed by performing a longitudinal cluster analysis of inflammatory mediators of known signaling mechanisms and general potential for inflicting or mitigating damage to the host. As shown with PCA, patients presenting a dynamic immune response associated with severity can be distinguished from survivors. Our study has limitations, primarily due to a small sample size of patient samples and attendant problems of limited generalizability and potential bias. Thus, we have emphasized the characteristics of individual cases as potentially representative of a subset of patients. Nevertheless, the results support a future study of a larger population either retrospectively and prospectively.

## Figures and Tables

**Table 1:** Summary of available data on patient history (red and bold font indicates Class III cases).

ID	Age/Sex	PMH	BMI	On-Set	1st Blood Draw	On ECMO	off ECMO	Outcome
**256**	**69/WF**		**27.1**	**dy0**	**dy4**	**dy4 **	**dy8**	**III; MOSF, died dy9**
258	61/WF		21.4	dy0	dy7	dy10	dy12	II; discharged **dy22**
259	62/WF		22.8	dy0	dy4	dy4	dy9	II; discharged **dy17**
260 *	**63/NAF**	**UTI, Diabetes, asthma, COPD**		**dy0**	**dy6 **	**n/a**	**n/a**	**III; died dy7**
270	26 NAF		31.2	dy0	dy3	dy3	dy8	II; discharged **dy26**
**280**	**30/M**		**32.1**	**dy0**	**dy3**	**dy4 **	**dy6**	**III; MSOF, DIC, died dy6**
**281**	**37/NAM**	**Diabetes, tobacco use disorder**	**32.9**	**dy0**	**dy3 **	**dy4**	**dy6**	**III; Hemorrhagic shock died dy6**
282	16/NAM	none	23.0	dy0	dy4	dy4	dy7	II; discharged dy30
293	18/HM	enlarged right axillary lymph node and smaller left axillary lymph nodes.	20.9	dy0	dy6	dy7	dy9	II; discharged from ICU **dy14**
306 *	65/WM	Enterococcus, Candidiasis of Mouth	28.8	dy0	dy5	dy5	dy11	II; transferred to SNIF **dy35**
319 *	**51/WF**	**Candidiasis of skin, nails, septicemia, pneumonia, herpes simplex**	**27.1**	**dy0**	**dy5**	**dy6**	**dy13**	**III; post ECMO complications died dy29**

*WF*, white female; *NAF/M*, Native American female or male; *HM,* Hispanic male; *PMH*, past medical history, not related to HCPS; *BMI*, body mass index; *Onset*, the day (dy0) when symptoms emerged according to the patient’s narrative; *1st blood draw*, when the patient was enrolled in the study relative to *Onset* typically day of admission. The day of the first blood draw is nominally defined as day 1 but has no real longitudinal time relevance. *ECMO*, extracorporeal membrane oxygenation; *UTI*, urinary tract infection; *MOSF,* multiple systems organ failure; DIC, disseminated intravascular coagulation; *SNIF*, Skilled Nursing Inpatient Facility; * bacterial coinfection.

## Data Availability

The data presented in this study are available on request from the corresponding author.

## Supplementary Materials

The following are available online at https://www.mdpi.com/article/10.3390/v13081597/s1: Figure S1, A plot of active and total uPA versus time for Class II and Class III; Figure S2, Pearson’s correlation test for PAI-1 versus L, N, IFN-γ, and TNF-α; Figure S3, Unsupervised comparisons of cytokines measured at distinct time points in each patient (270, 293, 258, and 259). Heatmap and cluster analysis of inflammatory mediators (cytokines, chemokines, growth factors, leukocytes, coagulation factors) measured in clinical patient plasma samples. A horizontal line was drawn in each heat map to indicate a suggested separation of inflammatory response patterns based on the cluster dendrograms and visual inspection of the heatmaps. The heat maps show pairwise Pearson’s correlations (color-coded: red, positive; blue, negative correlation) among inflammatory mediators sorted along both axes to emphasize object clusters using hierarchical clustering; Figure S4, Unsupervised comparisons of cytokines measured at distinct timepoints in each patient (256, 282, and 319). Heatmap and cluster analysis of inflammatory mediators (cytokines, chemokines, growth factors, leukocytes, coagulation factors) measured in clinical patient plasma samples. A horizontal line was drawn in each heat map to indicate a suggested separation of inflammatory response patterns based on the cluster dendrograms and visual inspection of the heatmaps. The heat maps show pairwise Pearson’s correlations (color-coded: red, positive; blue, negative correlation) among inflammatory mediators sorted along both axes to emphasize object clusters using hierarchical clustering; Table S1, Summary of medians and concentration ranges of the target cytokines measured in patient samples.
