# Supplementary material for: Longitudinal Assessment of Cytokine Expression and Plasminogen Activation in Hantavirus Cardiopulmonary Syndrome Reveals Immune Regulatory Dysfunction in End-Stage Disease
Source: Viruses. 2021 Aug 12;13(8):1597. doi: 10.3390/v13081597 (PMC8402847; doi:10.3390/v13081597)
Supplement: Supplementary file 1 [file viruses-13-01597-s001.zip › viruses-1284471-supplementary.pdf]

Supplementary Material

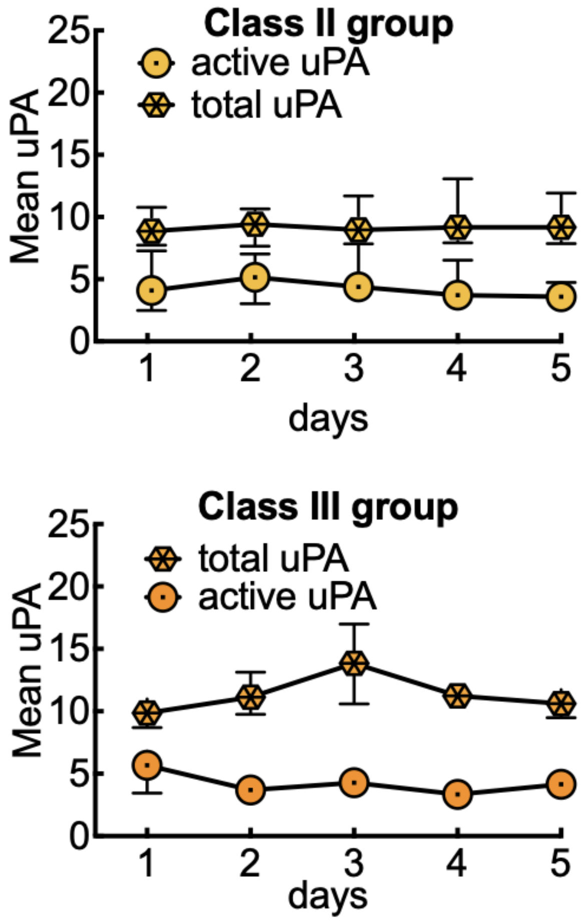

Figure S1. Plot of active and total uPA versus time for Class II and Class III.

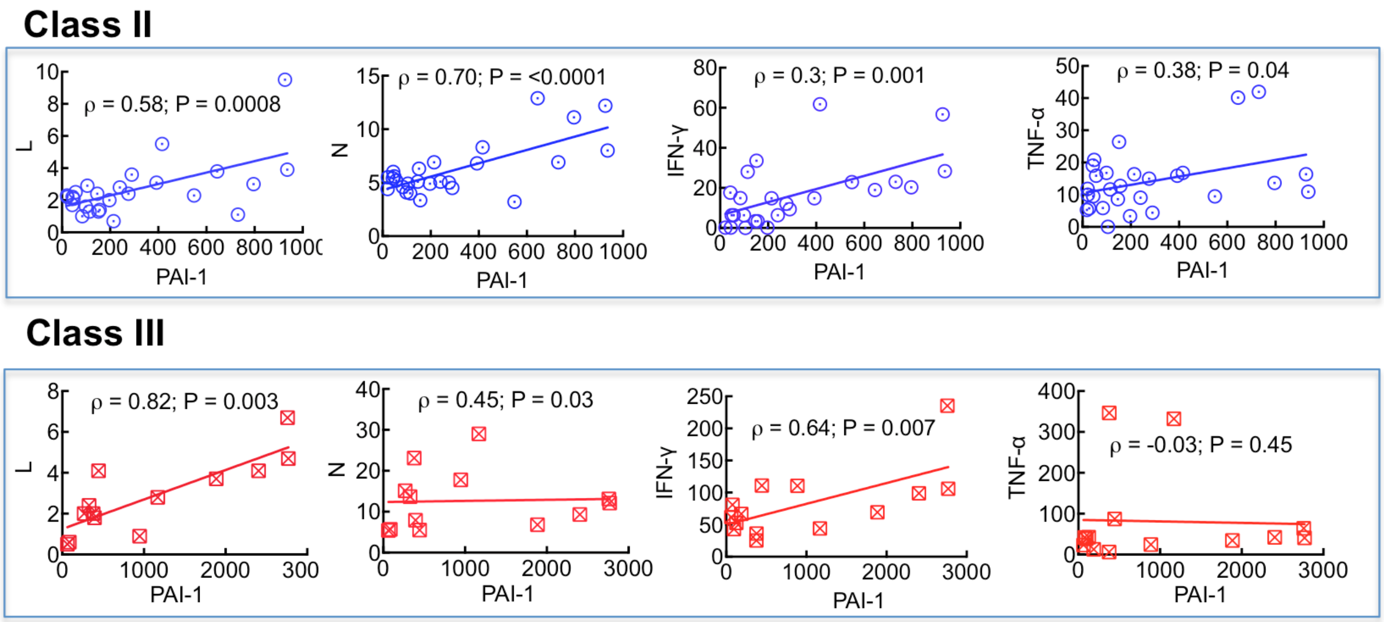

Figure S2. Pearson's correlation test for PAI-1 versus L, N, IFN-γ and TNF-α calculated and produced in GraphPad Prism version 9.1.2 https://www.graphpad.com/scientific-software/prism/.

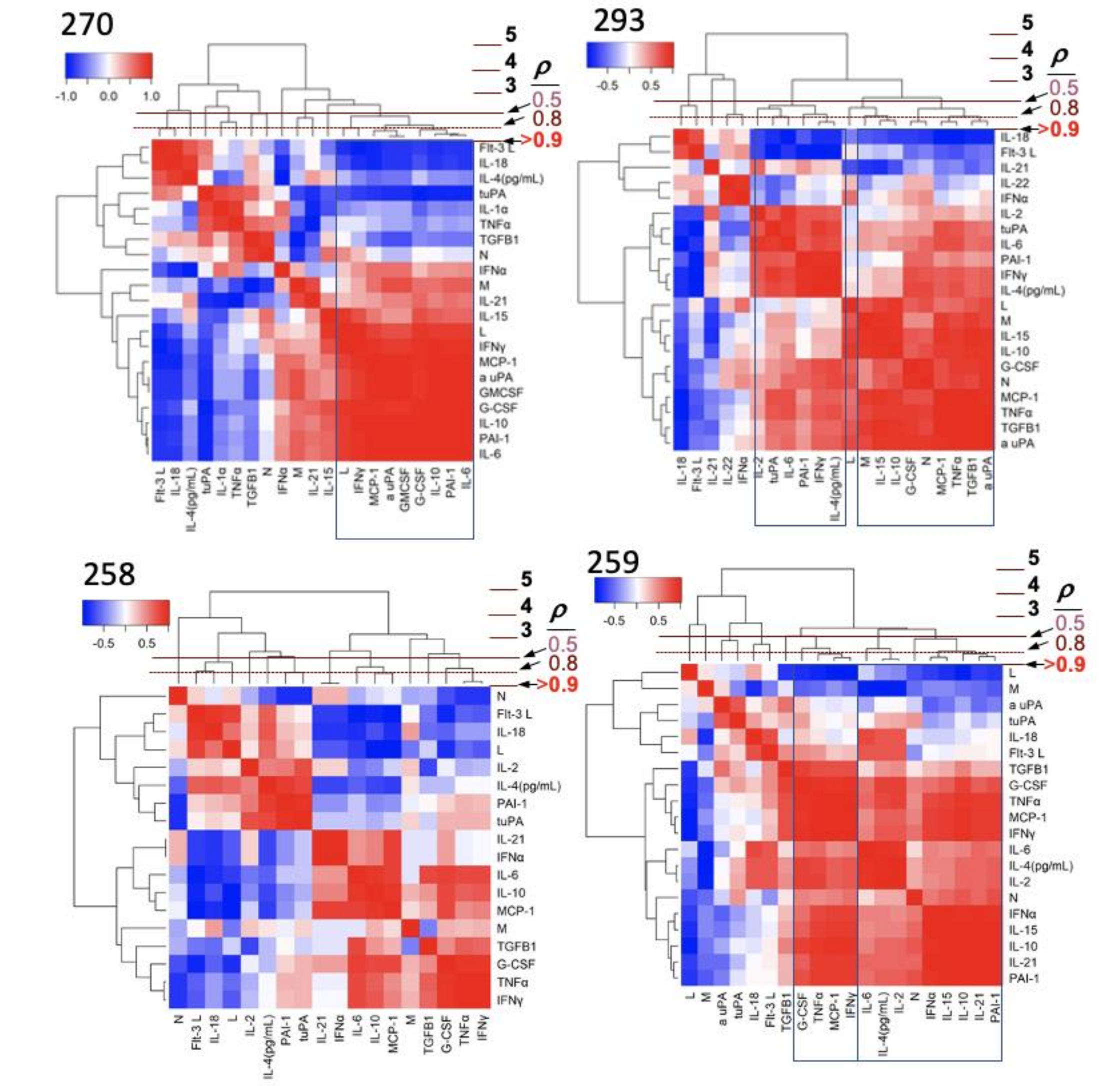

Figure S3. Unsupervised comparisons of cytokines measured at distinct time points in each patient (270, 293, 258, and 259). Heatmap and cluster analysis of inflammatory mediators (cytokines, chemokines, growth factors, leukocytes, coagulation factors) measured in clinical patient plasma samples. In each heat map, a horizontal line was drawn to indicate a suggested separation of inflammatory response patterns based on the cluster dendrograms and visual inspection of the heatmaps. The heat maps show pairwise Pearson's correlations (color coded: red positive and blue for negative correlation) among inflammatory mediators sorted along both axes to emphasize object clusters, using hierarchical clustering

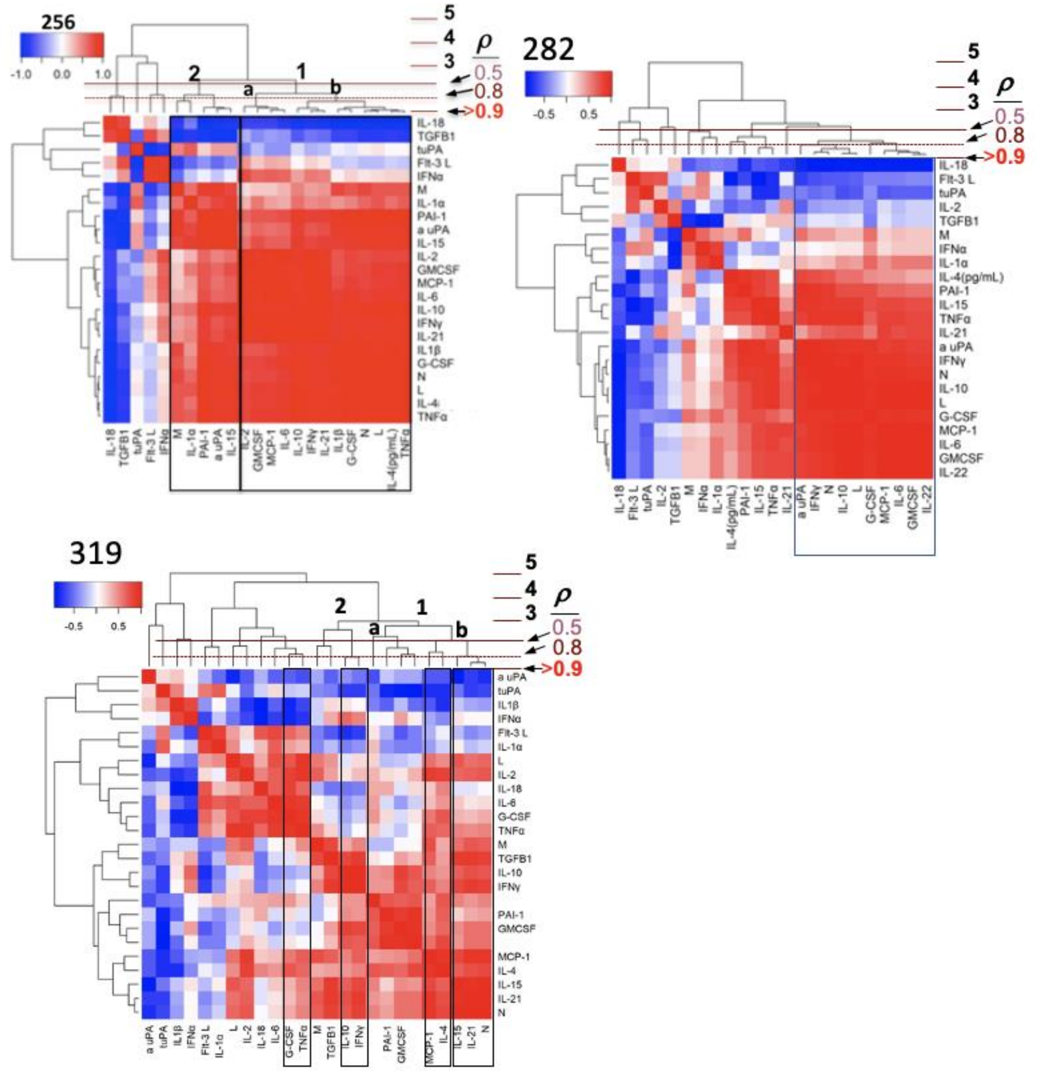

Figure S4. Unsupervised comparisons of cytokines measured at distinct time points in each patient (256, 282, and 319). Heatmap and cluster analysis of inflammatory mediators (cytokines, chemokines, growth factors, leukocytes, coagulation factors) measured in clinical patient plasma samples. In each heat map, a horizontal line was drawn to indicate a suggested separation of inflammatory response patterns based on the cluster dendrograms and visual inspection of the heatmaps. The heat maps show pairwise Pearson's correlations (color coded: red positive and blue for negative correlation) among inflammatory mediators sorted along both axes to emphasize object clusters, using hierarchical clustering

Table S1

**Cytokine, (ref. range, pg/mL)**	**III (n = 5)**[2] median (range) **(measures = 15)**[a]	**Fold change**[3]	**II (n = 6)** median, (range) **(N = 28)**[b]	**Fold change**	**I (n = 2)** (median, range) **(M = 8)**[c]	**Fold change**	**Controls (n = 5)** (median, range) **(N = 5)**[d]
IL-15 (2-5)	17.6 (273.8 – 6.8) (13)	**19.5**	2.7 (9.9 – 0.8) (18)	3	0.9 (1.5 – 0.4) (2)	-	nd
IL-18 (36.1-257.8)	863.4 (1512.5 – 355.3) (15)	3.6	380.8 (1050.1 – 261.0) (28)	1.6	863.4 (11512.5 – 355.3) (8)	3.6	238.5 (441.4 – 165.4) (5)
IL-12 p40 (<6)	11,093.1 (11,260.6 – 10,925.8) (2)		nd		nd		nd
IL-12 p70 (<6)	nd		nd		nd		nd
IL1β (<36)	9.6 (297.1 – 1.0) (9)		nd		nd		nd
TNF-α(<22)	40.8 (346.5 – 6.2) (15)	11.0	12.7 (41.9 – 3.3) (27)	3.4	3.7 (5.8 – 2.8) (8)	-	nd
**IFN-γ(<5)**	66.8 (235.4 – 20.3) (15)	**66.8**	14.9 (61.8 – 0.01) (24)	14.9	1.0 (1.8 – 0.2) (2)		nd
IL-1α (0.4-1.4)	4.5 (242.1 – 1.2) (10)	2.8	1.6 (2.1 – 0.6) (4)		nd		nd
IFN-α (**X**)	3.0 (149.4 – 0.8) (11)	1.4	1.9 (8.0 – 0.3) (23)	0.9	2.2 (3.9 – 0.3) (6)	-	nd
IFN-β (**X**)	0.010 (0.01 – 0.01) (2)	0.03	0.3(0.7 – 0.01) (2)	-	nd		nd
IL-2 (<12)	4.8 (393.8 – 1.7) (14)	2.0	3.3 (6.1 – 0.6) (24)	1.4	2.8 (6.1 – 1.1) (8)	1.2	2.3 (4.6 – 1.7) (3)
IL-6 (0 – 0.4)	622.0 (27,651.1 – 11.5) (15)	**248.8**	51.6 (1,845.5 – 12.3) (28)	20.6	6.0 (17.4 – 5.4) (8)	2.4	2.5 (2.5-2.5) (1)
IL-4 (<5)	181.8 (330.0 – 35.4) (15)	4.8	59.0 (124.5 – 28.2) (28)	1.5	29.3 (44.2 – 27.7) (8)	0.7	38.2 (41.9 – 24.5) (5)
**IL-10 (<18)**	106.9 (300.6 – 13.5) (14)	**9.4**	45.2 (977.1 – 5.1) (26)	4.0	11.490 (23.4 – 2.6) (4)	-	nd
**TGF-β1 (8,289 - 63,416)**	45,959.6 (122,924.2 – 5,499.9) (15)	3.5	16,709.1 (27,010.9 – 9,126.7) (28)	1.3	11,756.6 (20,649.4 – 6,888.2) (8)	0.9	13,339.3 (24,559.0 – 10,907.9) (5)
IL-21 (**X**)	16.6 (31.3 – 1.9) (15)	**16.6**	3.7 (19.0 – 1.0) (23)	3.7	1.0 (1.9- 0.07) (5)	-	nd
GM-CSF (3-122.)	1.90 (236.6 – 0.14) (11)	2.5	0.760 (3.3 – 0.2) (6)	-	nd		nd
IL-22 (**X**)	39.0 (387.7 – 14.4) (5)	1.0	9.4 (17.9 – 0.5) (7)	0.2	11.7 (19.8 – 6.1) (3)	0.3	38.3 (38.3 – 38.3) (1)
IL-13 (<5)	166.9 (10,757.9 – 120.0) (6)	1.4	64.3 (166.9 – 64.3) (3)	0.5	nd		120.0 (120.0 – 120.0) (1)
IL-25 (**X**)	13,752.9 (14,171.0 – 104.9) (3)	**302**	45.5 (104.9 – 12.3) (3)	-	nd		nd
MCP1/CCL2 (2-48)	2,266.5 (16,796 – 305.1 (15)	**16.0**	652.1 (3,365.1 – 105.2) (28)	4.6	360.9 (701.6 – 222.2) (8)	2.5	139.9(204.8 – 90.5) (5)
Flt-3 Ligand (**X**)	150.6 (479.3 – 55.3) (15)	1.8	75.4 (262.6 – 17.3) (28)	0.8	64.7 (113.0 – 49.7) (8)	0.7	85.5 (134.1 – 76.3) (5)
G-CSF (<1.5)	350.1 (1961.6 – 91.9) (14)	6.6	91.6 (459.4 – 25.1) (28)	1.7	57.9 (73.4 – 27.3) (8)	1.0	52.9 (72.5 – 25.7) (5)

1 http://www.bio-rad.com/webroot/web/pdf/lsr/literature/Bulletin_6029.pdf

IFNγ is superior to TNF◎ in eliciting uPA activity. 2. **n** refers to the number of cases, **N** refers to the number of data points from available serial samples. **a**. 15 serial samples available from 5 cases: 256 (4 samples), 318 (6 samples), 260 (2 samples), 280 (2 samples) and 281 (1 sample). **b.** 28 serial samples from 6 cases **c.** 8 serial samples from cases 285 and 318. **d**. 5 samples from 5 control samples. Fewer measures than sample size is due to undetected cytokine expression in some samples. **3.** Fold changes were calculated based on the median expression in healthy controls, Class I or II, depending on detection limits.
